# Comparative experimental anesthesia efficacy study of epidural injection at the sacrococcygeal space using ultrasound guidance versus blindness technique in Egyptian donkeys (*Equus asinus*)

**DOI:** 10.1186/s12917-025-04475-8

**Published:** 2025-02-07

**Authors:** Mohamed A. Hamed, Hazem Hamouda, Mohammed Elmetwally, Foad Farrag, Mohamed Abdo Rizk, Mohamed M. A. Abumandour, Yamen Mohammed Hegazy, Alaa Samy

**Affiliations:** 1 Department of Surgery, Anesthesiology, and Radiology, Faculty of Veterinary Medicine, Aswan University, Aswan, Egypt; 2 Department of Anatomy and Embryology, Faculty of Veterinary Medicine, Aswan University, Aswan, Egypt; 3 Department of Theriogenology, Faculty of Veterinary Medicine, Mansoura University, Mansoura, Egypt; 4 Department of Anatomy and Embryology, Faculty of Veterinary medicine, Kafrelsheikh University, Kafrelsheikh, Egypt; 5 Department of Internal Medicine and Infectious Diseases, Faculty of Veterinary Medicine, Mansoura University, Mansoura, 35516 Egypt; 6 Department of Anatomy and Embryology, Faculty of Veterinary Medicine, Alexandria University, Alexandria, Egypt; 7 Department of Animal Medicine, Faculty of Veterinary Medicine, KafrelsheikhUniversity, 33511 Kafrelsheikh Egypt; 8 Department of Clinical Sciences, College of Veterinary Medicine, King Faisal University, Al-Ahsa, 31982 Saudi Arabia; 9 Department of Surgery, Anesthesiology, and Radiology, Faculty of Veterinary Medicine, Mansoura University, Mansoura, 35516 Egypt

**Keywords:** Blind caudal epidural injection, Egyptian donkey, Sacrococcygeal space, Ultrasound-guided caudal epidural injection

## Abstract

The study investigates the effectiveness of blind and ultrasound-guided epidural injections in Egyptian donkeys in the sacrococcygeal region, a topic of limited research, by comparing and assessing their onset in 20 healthy adult donkeys of both sexes. In the first group, we used ten cadaver donkeys that were humanely euthanized. In the second group, we used 10 live, healthy adult donkeys to assess the efficacy and time of analgesia onset for blind and ultrasound-guided epidural injections at the sacrococcygeal region. Cadavers were randomly designated to blind (*n* = 5) or US-guided (*n* = 5) epidural injections at the sacrococcygeal region in sternal recumbency in donkeys using Methylene Blue (1%) as a diagnostic marker for caudal epidural injection. The injection criteria were comparatively assessed between the two techniques of injection. Our findings comparing the blind and US-guided injection approaches showed substantially higher needle accuracy penetration to comparatively assess these two injection techniques. Both injection difficulties and trial numbers were significantly greater in blind techniques as opposed to US-guided procedures. US-guided injection procedures revealed the effectiveness of the time needed for perfect placement of the needle was significantly less than with a blind one. A shorter time for the onset of analgesia was achieved with the ultrasound-guided method, although the difference was not statistically significant (*P* < 0.09). In conclusion, the ultrasound-guided technique of the epidural injection provided a number of benefits over the blind one, including the capacity to directly view the needle and distribute local anesthetic and avoid unintentional vascular damage, in comparison with traditional blind techniques. Our study aims to enhance the accuracy and safety of epidural anesthesia injection at the sacrococcygeal space in Egyptian donkeys using ultrasound guidance in the veterinary surgical field.

## Introduction

Donkeys, domesticated *Equidae* members, were utilized in underdeveloped countries for draft and riding, primarily in rural areas, making them particularly crucial in developing nations with large numbers of donkeys [1–3]. Egypt’s donkey population has decreased from 3.2 million in 2014 to one million due to factors such as Chinese market demand, overexploitation, industrialization, and increasing Egyptian donkey demand for preparation and industrialization [4–6].

Ultrasound imaging techniques enable accurate needle placement and real-time monitoring of local anesthetic solution administration [7, 8]. The advantages of this approach include enhanced nerve block effectiveness, faster onset times, and reduced local anesthetic solution needed for effective block production [9, 10]. In contrast to traditional blind techniques, which may fail because of an uneven distribution of local anesthetic, US can confirm the distribution of local anesthetic around the target nerve, which is a major advantage for peripheral nerve block procedures [11]. There are numerous published data points that are related to caudal epidural anesthesia, but they are mainly focused on studying the effects of anesthesia drugs [12, 13]. However, there is a shortage of information about the method of anesthesia technique rather than the blind caudal epidural anesthesia injection method [14]. Caudal epidural anesthesia is a technique used in surgical interventions to inject a local anesthetic into the epidural space, preserving the pelvic limb’s function, especially in large animal patients who are standing sedated during the procedure [15–19]. The sacrococcygeal epidural injection using a blind technique can be challenging, particularly in small animals with high body condition scores, due to the presence of abundant subcutaneous fat [20]. Caudal epidural analgesia is frequently utilized on standing sedated animals during surgical operations since it preserves the mobility of the pelvic limbs [17, 18].

Excellent accuracy can be achieved by injecting the horse cervical nerve roots using ultrasound guidance. The injection is deposited in direct contact with up to 75 of the C3-C8 cervical nerve roots [21]. In seven obese dogs, the location of the needle insertion for the lumbosacral epidural injection was determined by ultrasound imaging [22, 23]. Ultrasound guidance is frequently used in human anesthesia to guide the placement of caudal epidural needles [24]. Ultrasound-guided epidural injection technique is recently introduced in the veterinary surgical science [11]. The success rate of anesthetists administering epidural anesthesia is increased by ultrasound evaluation prior to the injection, which also shortens their learning curve [25]. A recent parturient study found that prepuncture ultrasonographic evaluation of the lumbar region was linked to a notably lower number of puncture attempts and a quicker process time; these benefits were especially noticeable in individuals who were obese. The first-attempt success rate for obese patients under ultrasound guidance was 92%, compared to 44% when utilizing a traditional blind approach [26]. Though the relevance and value of this image-guided process for fewer skilled veterinary operators in comparison to the traditional landmark blind method had not been examined, it has potential value [20].

According to the authors’ knowledge, no veterinary study has taken into account administering an ultrasound-guided epidural injection to the donkey’s sacrococcygeal region. Many ultrasound-guided methods define needle insertion at the cisterna magna in horses for CSF collection [27], among the first and second cervical vertebrae [28], and in the lumbosacral space [29]. One paper describing canine sacrococcygeal epidural injection aided by ultrasonography [20]. It was hypothesized that the utility of ultrasound guidance to optimize needle location and the viewing of sacrococcygeal space and adjacent structures would enhance the technique’s safety and success rates when compared to blind techniques. The goal of this investigation was to evaluate the efficacy of blind and ultrasound-guided epidural injection in the donkey’s sacrococcygeal region in cadavers and clinical cases, as well as to explain the ultrasonographic anatomy of the sacrococcygeal region in donkeys. In the end, the current findings were compared to the formerly published data about the epidural injection in the sacrococcygeal area in the different domestic species.

## Materials and methods

### Animals collection and study design

This study was conducted on a total of twenty adult Egyptian donkeys (*Equus asinus*) of both sexes (sexes not recorded) and weighed (mean ± SD) 130.4 ± 3.59 kg. To ensure the validity of these donkeys to the experiment, the musculoskeletal system of these donkeys was examined physically, radiographically, and ultrasonographically. The collected donkeys used were free from any vertebral column anatomical abnormalities. The Egyptian donkeys were grouped into two experimental groups. In the first group, we used ten cadaver donkeys that were humanely euthanized. In the second group, we used 10 live, healthy adult donkeys to assess the efficacy and time of analgesia onset for blind and ultrasound-guided epidural injections at the sacrococcygeal region. These donkeys were obtained directly from their respective owners, and the informed consents from the owners have been duly obtained. The anatomical terms were applied according to **Nomina Anatomica Veterinaria** [30].

### Cadaveric study

This study was conducted on ten donkey cadavers as soon as possible (1–2 h) after a humane euthanasia by a rapid intravenous injection of thiopental sodium (1-g vial; EPICO, Egypt) at a dose of 35 mg/kg BW, as illustrated by Hamed, et al. [31]. Cadavers were randomly subdivided according to the method of epidural injection into blind (*n* = 5) or US-guided (*n* = 5) epidural injections at the sacrococcygeal region. In this study, an area extending from the initial third of the tail to the lumbosacral area was clipped and prepared aseptically. Then, we applied the blind epidural injection and ultrasound-guided epidural injection technique at the sacrococcygeal space in the donkeys as follows:

#### Blind epidural injection

**Fig. 1:**
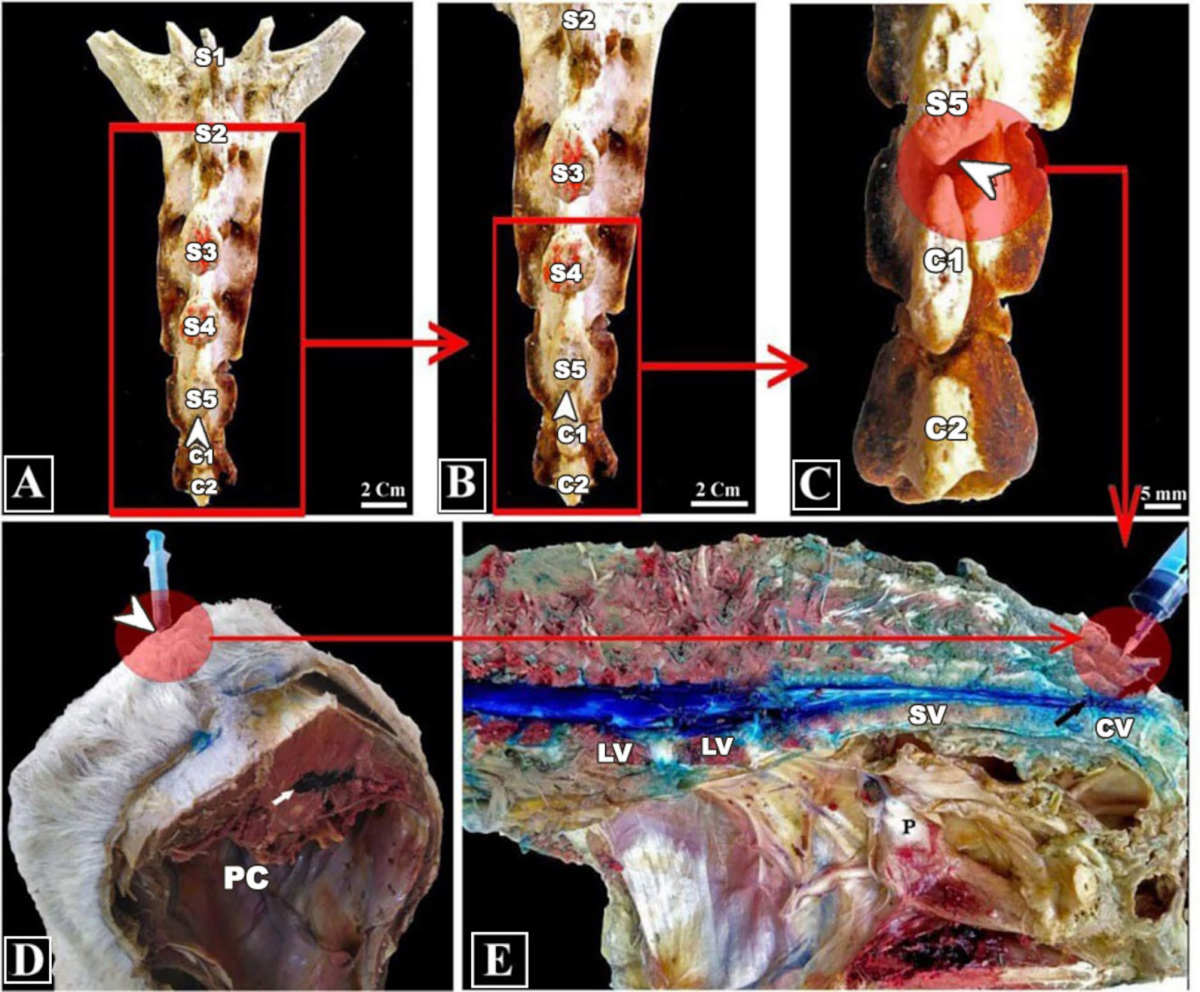
Gross Anatomical of the pelvic region of the Egyptian donkey. View (**A**, **B**, and **C**) showing the sacral vertebrae and coccygeal vertebrae in addition to sacrococcygeal space (arrow heads); View (**D**) showing cross section of body in which appeared the site of injection of methylene blue (arrowhead) and the methylene blue inside the vertebral canal at the level of lumbar vertebrae (arrow); and View (**E**) showing the sagittal section of pelvic region. **Abbreviations**: the pelvic cavity (PC); lumbar (LV); 1st (S1), 2nd (S2), 3rd (S3), 4th (S4), and 5th (S5) sacral vertebrae (SV); 1st (C1) and 2nd (C2) coccygeal vertebrae (CV) and site of injection at the sacrococcygeal space (arrow)

Blind injection was carried out by detecting the sacrococcygeal space and inserting a 20-gauge needle (Med, Eldawlia ico, Egypt) into the space, precisely aiming to create a successful injection. Following the surgeon’s confirmation that the right position had been achieved, 6 mL of 1% methylene blue solution was injected into the spinal canal at the sacrococcygeal space (Fig. 1).

#### U.S-guided epidural injection

An ultrasound machine (Mindray DP-2200Vet., PR China) with a 7.0 to 10.0 MHz convex probe was used to carry out US-guided injection in both transverse and longitudinal planes. Initially, the transducer was positioned transversely across the sacrum caudal area. The sacrococcygeal space was found and viewed by moving the probe craniocaudally to the vertebral column. The needle was punctured via ultrasound guidance. The needle tip proceeded toward the sacrococcygeal area till it approached the spinal canal floor while the transducer was in place. On ultrasonography, the needle tip placement with relation to the sacrococcygeal space was examined, and if required, adjustments were made. The dye’s entry into the area was seen as an anechoic, fluid wave on the ultrasonography image. After epidural injection, either blindly or U.S. guided, anatomic dissection of the preserved specimens was performed with great care, documented, and photographed in order to look for the existence of methylene blue in the vertebral canal. Following dissection, the existence of methylene blue in the spinal canal was seen as evidence of a successful injection (Fig. 1E).

### In vivo study

**Fig. 2:**
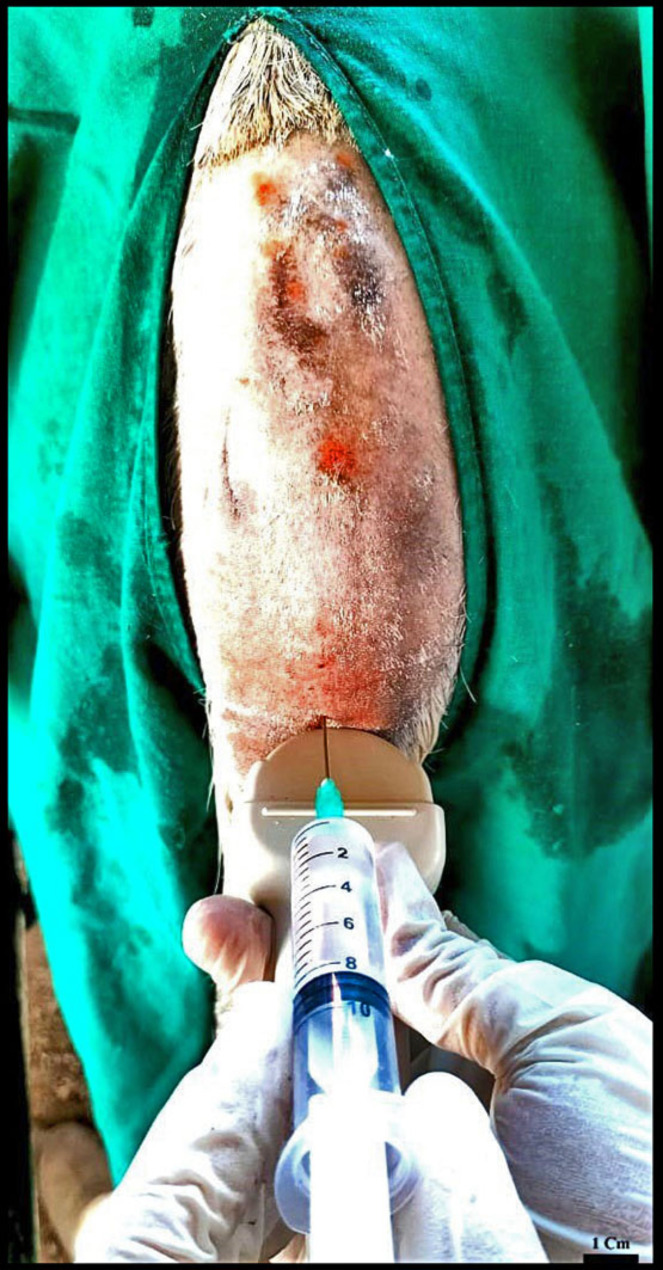
Gross image describing the Ultrasound-guided sacrococcygeal epidural injection of lidocaine hydrochloride in Egyptian donkey; the ultrasound probe was positioned transverse to the vertebral column

Ten healthy adult donkeys were employed to evaluate the effectiveness and precision of blind and ultrasound-guided epidural injections at the sacrococcygeal region. All donkeys were kept in stocks for adequate restraint, and then aseptic cleaning and shaving were done in the examining area. Firstly, sedation of donkeys was induced via intravenous administration of a combination of acepromazine (Vetranquil, 0.02 mg/kg body weight intravenously) and detomidine (Detogesic, 0.01 mg/kg body weight intravenously) with a 15-minute interval. Then, a subcutaneous anesthetic injection of 2 ml of lidocaine HCl was utilized. The precise location was determined by palpating the indentation between the sacrum and the first caudal vertebra and moving the tail up and down. A thirty-five degree angle with the median plane was formed when the 20G needle was placed into the skin. To verify that the needle was positioned correctly, the hanging drop technique was utilized to identify negative pressure, and the injection went through without resistance. After negative aspiration, 6 mL of 2% lidocaine hydrochloride (Lidocain Ccl, B. Braun Melsungen AG, Germany) was injected epidural into the sacrococcygeal area (Fig. 2). The blind or ultrasound-guided approach was considered successful when the perineal region’s cutaneous sensibility was completely lost.

Sensation was confirmed at the perineal region with a pinprick test using a 22-gauge (2.5 cm) needle prior to lidocaine injection (baseline) and at two-minute intervals thereafter until sensation was abolished. The needle was inserted into the skin, and the animal’s reaction to this stimulus was observed. Analgesia was deemed effective when the animal accepted the skin puncture and did not react to pricking. When the animal shifted its head, neck, and/or trunk to preclude the unpleasant stimulation of the needle, analgesia was deemed ineffective. The time (in minutes) spent between the injection of the local anesthetic and the lack of sensation was regarded as the start of local analgesia. Three days following injection, the donkeys were observed to check for any possible complications, including infection, hematoma, or neurological issues.

### Injection criteria

The injection criterion evaluation was the responsibility of individual practitioners. A subjective grading method for the ease of accurate needle penetration, the difficulty of injection, the number of tries, and the performance time was used to evaluate and score the expert’s estimated confidence at injection (Table 1), according to **El-Shafaey**, et al. [32]. In both cadaveric and live animal studies, injection criteria were applied. Skilled anatomists, sonographers, radiologists, and anesthetists assessed and judged the injection criteria’s effectiveness. The epidural injection procedures for both the cadaveric and live animal experiments were carried out by a skilled anesthetist.

**Table 1:** Donkeys’ subjective assessment of sacrococcygeal epidural injection via a descriptive scale

Parameters	Score description
**Correct penetration**	0 = Poor, out of the target joint capsule 1 = Good, in the way but not entered the target joint capsule 2 = Excellent, in the target joint capsule
**Needle localization**	0 = Poor, the needle not clearly localized 1 = Good, the needle localized but not in target site 2 = Excellent, the needle localized in target site
**Difficulty of injection**	0 = Difficult, several attempts with low confidence 1 = Moderate, several attempts until successful injection 2 = Easy, immediate and confident injection
**Number of trials**	0 = more than three attempts, 1 = two to three attempts, 2 = One attempt
**Performance time (min)**	0 = 11–15, 1 = 6–10, 2 = up to 5 min
**Onset of analgesia (mins)**	1= delayed( more than 20 min), 2 = moderate (11–20), 3= Rapid (5–10)
**Duration of analgesia (mins)**	1 = Short duration (less than 30 min), 2 = moderate (30–60 min), 3 = long duration (more than 60–90 min)

### Statistical analysis

The statistical software package GraphPad Prism (GraphPad Prism for Win. Version 5.0, GraphPad Software Inc., USA) was used to conduct the statistical analysis. All scores were expressed as median (minimum–maximum). A pairwise comparison between the two injection criterion scores (non-parametric data) was carried out using the Mann Whitney U test. When *P* < 0.05, significance was deemed to exist after applying the bonferroni correction for multiple comparisons between variables.

## Results

### Cadaveric study

In a cadaveric investigation, US-guided epidural injection at the sacrococcygeal region proved to be a practical, dependable, and precise method. Through cadaver dissection, it was verified that the sacrococcygeal space was correctly identified in every cadaver and dyed in every case (Fig. 1E).

**Fig. 3:**
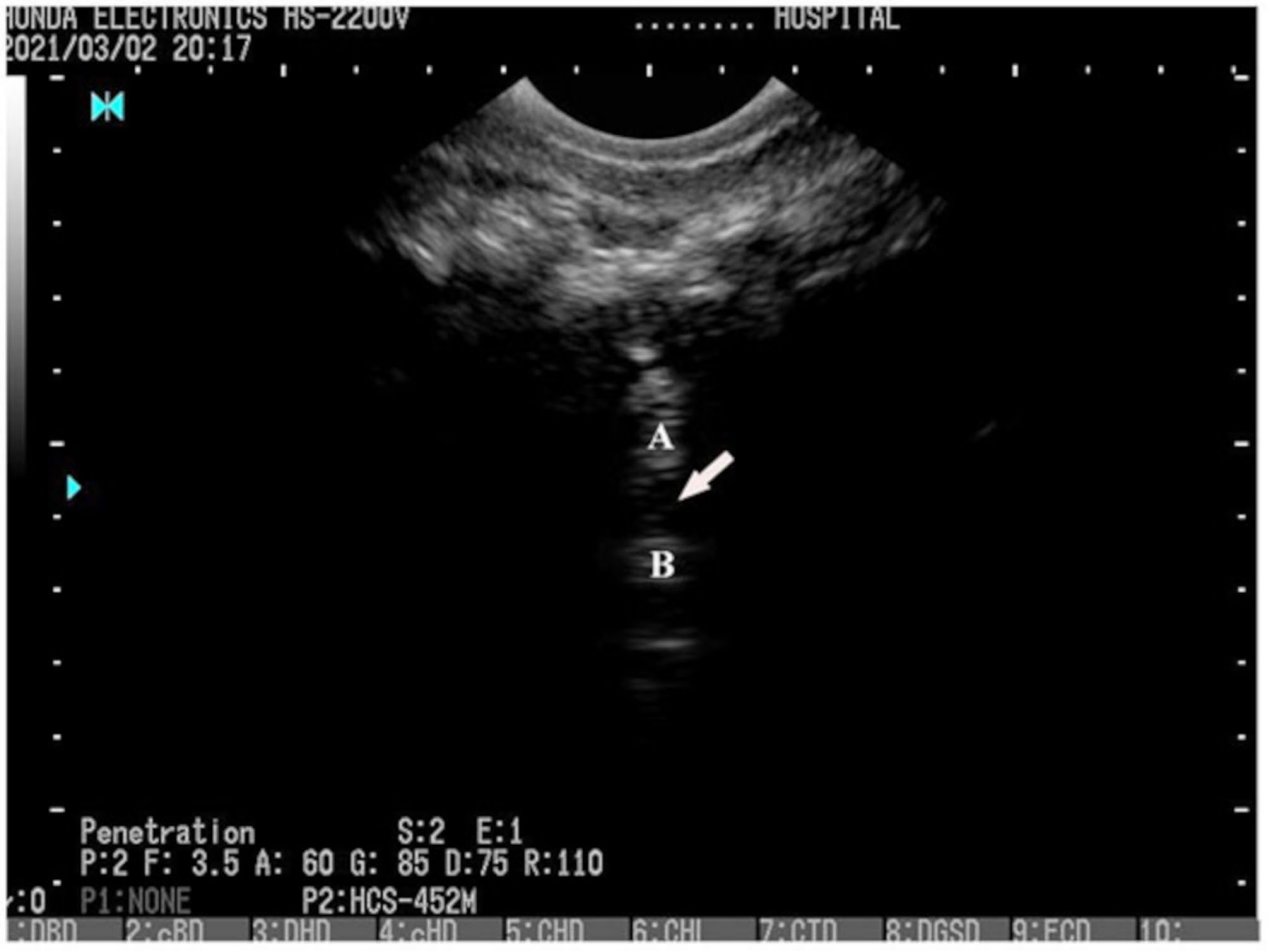
Ultrasonographic image of the in Egyptian donkey showing the sacrococcygeal space (arrow) as a hypoechoic circular area delimited dorsally and ventrally respectively by the arch (**A**) and body (**B**) of the first caudal vertebra. An ultrasound machine (Mindray DP-2200Vet., PR China) with a 7.0 to 10.0 MHz convex probe was used to perform US-guided injection in transverse plane

On the ultrasonogram, the caudal portion of the sacrum served as the prominent landmark for transverse transducer location with respect to the donkey vertebral column. The sacral crest appeared in the image as a thin, hyperechoic vertical structure, and two perpendicular hyperechoic lines on either side of it depicted the caudal sacral processes. Caudally to this site, the sacrococcygeal space, which represents the body and arch of the first caudal vertebra, was found to be a circular hypoechoic region. It was defined by hyperechoic structures that produced distal acoustic shadowing (Fig. 3). The floor of the spinal canal was indicated by the thin, straight, hyperechoic line with distal acoustic shadowing that was found underneath the epidural space. The needle was pushed into the epidural space through the opening between the sacrum and the dorsal lamina of the first caudal vertebra until it was visible that it touched the vertebral canal floor. In all of the dissected cadavers, no blood vessel was found to be stained with the blue dye, indicating that no vascular components had accidentally entered the body.

Entirely, comparing the blind technique to the US-guided epidural injection at the donkey’s sacrococcygeal region, there was generally a significant increase (*P* < 0.05) in the injection parameters (Table 2). Analysis of injection parameters between US-guided and blind injection procedures showed that the US-guided methods had substantially higher needle accuracy penetration than the blind methods. Injection difficulties and trial count were significantly higher in blind techniques compared to US-guided procedures.

**Table 2:** Effect of correct penetration, needle localization, difficulty of injection, number of trials, performance time on the injection scores for sacrococcygeal epidural injection in donkeys

Parameters	Blind	Ultrasonography	*P* value
**Correct penetration**	0 (0–1)^b^	2 (1–2)^a^	0.001***
**Needle localization**	0.5 (0–1)^b^	2 (1–2)^a^	0.002***
**Difficulty of injection**	1 (0–1)^b^	2 (1–2)^a^	0.0003***
**Number of trials**	0.5 (0–1)^b^	2 (1–2)^a^	0.003***
**Performance time (min)**	0 (0–1)^b^	1 (1–2)^a^	0.002***

^a^ and ^b^ Medians and ranges with different superscript letters at the same column are significantly different at *P* < 0.05

The frequency of trials required for an efficient injection is higher in the blind technique in comparison with US-guided injection techniques (0.5 vs. 2, respectively) (Table 2). Nonetheless, US-guided injection techniques required a shorter performance time (3 min vs. 5 min, respectively) for accurate needle placement than the blind method.

### US-guided approach required a short time for the onset of analgesia

Live donkeys exhibited good tolerance to both blind and ultrasound-guided methods of epidural injection at the sacrococcygeal region. Visualization of the identical structures seen in the cadavers was achievable using the ultrasound-guided approach. The appearance of the epidural space and surrounding structures in living and dead animals did not differ from one another on ultrasonography.

In ultrasonography, visualization of the needle tip within the vertebral canal was also achievable in all cases. Aspiration that was negative verified the correctness and omission of vascular structures. The local anesthetic solution was injected gradually and was always visible in real time after the needle tip was inside the sacrococcygeal region (Fig. 2). All animals experienced a loss of analgesia in the perineal regions within five to ten minutes. During or after this procedure, there were no visible or ultrasonographic defects found.

In the ‘blind’ technique, the bony characteristics could be identified in every instance. The needle was proceeded to the sacrococcygeal area after aspiration was tried prior to each injection, and the needle was removed and reinserted till aspiration proved negative. Desensitization of the perineal region was attained in 3/5 cases, and the onset of analgesia began to take effect between 15 and 20 min later. Each donkey recovered calmly from the trials and displayed no signs of any neurological abnormalities or evidence of nerve harm. Compared to the blind procedure, the ultrasound-guided approach resulted in a shorter time for the onset of analgesia, but it was non-significant (*P* < 0.09). The duration of analgesia in both groups (up to 75 min) did not differ significantly (Table 3).

**Table 3:** Effect of blind and US injection scores for sacrococcygeal epidural injection in donkeys on the onset of analgesia and duration of analgesia

Parameters	Blind	Ultrasonography	*P* value
**Onset of analgesia**	2 (2–3)^b^	3 (3)^a^	0.09
**Duration of analgesia**	3 (2–3)^b^	3 (2–3)^a^	0.99

^a^ and ^b^ Medians and ranges with different superscript letters at the same column are significantly different at *P* < 0.05

## Discussion

The field of veterinary anesthesia is persistently pursuing suitable alternative epidural injections, which present a higher chance of success due to needle placement accuracy, dependability, and safety [24, 28, 33]. Finding the best imaging modality for epidural administration in veterinary medicine is a topic of controversy. As a result, the purpose of this investigation was to compare the viability and application of US-guided injection techniques versus blind approaches for epidural injection in the sacrococcygeal area of donkeys. This is one of the first studies that compares and explains the “blind” and ultrasound-guided methods for administering epidural injections at the sacrococcygeal space in Egyptian donkeys, as far as we know.

In a cadaveric investigation, US-guided epidural injection at the sacrococcygeal region proved to be a practical, dependable, and precise method. The sacrococcygeal epidural space was observed in the present investigation using ultrasonography in cadavers and clinical donkeys. It was identified as a hypoechoic circular zone situated caudal to the sacrum, which was defined by the hyperechoic bony components that comprised the first caudal vertebra. This look was comparable to the sacral hiatus that has been reported in canines [20, 33], who stated that the sacrococcygeal epidural space can be found by using the median sacral crest and the caudal sacral processes as helpful starting markers. Furthermore, the transverse approach provided the ultrasonographer with a small window dorsally between the last sacral vertebrae and the 1st caudal vertebrae, enabling the ultrasonographer to photograph the vertebral canal.

The surgical technical skills of sacrococcygeal space piercing can be difficult to educate to rookie operators, as the feedback provided by the sensation of tissue layers during needle injection can be challenging to communicate. Before carrying out a procedure in a clinical context, training on a cadaver is thought to have the benefit of lowering a novice’s anxiety and offering nontheoretical, practical hands-on training [34–36]. In this study, the cadaver model consisted of fresh cadavers, which were thought to be an appropriate tool for training and learning about piercing a needle into the sacrococcygeal space before clinical practice. Furthermore, the precise in vivo sensation of tissue layers, animal responses (such as twitching of the tail or popping sensation entering the sacrococcygeal space), and the potential existence of CSF and blood could only be felt and experienced on a live anesthetized donkey, as reported by **Etienne**, et al. [37].

Gross dissection of cadavers was useful in this study to establish an appropriate technique for puncturing the sacrococcygeal space in donkeys by identifying anatomical landmarks, placing needles correctly, checking for the presence of methylene blue in the vertebral canal, and assessing potential damage to surrounding structures. Following ultrasound, cadaveric dissection verified that each case’s sacrococcygeal area had been precisely located and stained. There was no evidence of vascular injury in any of the dissected cadavers since no blood vessel was found to be stained with the blue dye. These findings were similar to those previously described in canines, donkeys, and Egyptian buffaloes [37–39].

Ultrasound is generally regarded as a highly helpful instrument for consists of CSF on the atlanto-occipital location in standing horses. Because, it reduces the attempt numbers, speeds up the process, and minimizes blood contamination and damage [28, 40]. To achieve the puncture while utilizing a blind technique based primarily on anatomical markers, the horse must be in a symmetrical and static position. Accurate site localization and puncture are made possible by ultrasonography [29]. According to our investigation, US-guided injections had a noticeably higher accuracy rate than blind injections. This may be related to the viability of US-guided injection, which visualizes the needle tip and fluid flow during epidural injection, allowing the needle to be directed to the sacrococcygeal area while avoiding important structures. Our results aligned with those described in horses [27, 41].

The objective of the live study was to eradicate post-mortem alterations in cadaveric specimens and exhibit crucial factors, such as the attitude, pain, and conduct of the living animals throughout the injection. In live donkeys, both blind and ultrasound-guided methods of epidural injection at the sacrococcygeal region were tolerated effectively. In both living and dead animals, there were no variations in the ultrasonographic features of the surrounding structures and the epidural space. Aspiration that was negative verified the correctness and omission of vascular structures. Our findings are consistent with those described in the horses and buffalos [36, 39].

In this study, US-guided sacrococcygeal space injection produced superior results than the blind approach for all injection metrics, as well as increased specificity. The optimal needle position inside the sacrococcygeal space, the positive non-invasive visualization of the spinal canal, and the high-quality ultrasound images improve accuracy and shorten the time (3 min vs. 5 min, respectively) and number of trials needed for epidural injection. These findings align with those reported by [35, 42]. On the contrary, when US-guided techniques were used to inject the scapulohumeral joint (SHJ), bicipital bursa (BB), and infraspinatus bursa (IB) in horses, the regular time was noticeably longer. It was believed that this was directly linked to the operator’s inexperience [43].

The blind sacrococcygeal epidural injection technique is difficult to use since it depends on the palpation of surface anatomic indicators [32]. In the current investigation, a shorter time for the onset of analgesia was achieved with the ultrasound-guided method, although the difference was not statistically significant. This could be because it is challenging to determine the precise anatomical site where the needle should be inserted, which could result in improper insertion of the needle and inadequate injection [40, 41]. Therefore, the current study offers a foundation for reference for refining injection techniques for sacrococcygeal epidural injection in donkeys using US-guided sacrococcygeal epidural injection.

This study has limitations that should be noted. The first is that an ultrasound technique’s effectiveness depends on its operator. Being proficient in ultrasound is a unique ability that takes training and skill. The study’s small sample size of animals is the second drawback. The effect of age, sex, body condition score, and body weight of each animal on the size and contents of the epidural space is the third drawback. To confirm the usefulness of the procedure on a larger sample size in clinical cases and to arrive at a definitive conclusion, future investigations should take into account the limitations of this study.

In conclusion, US-guided epidural injection at the sacrococcygeal space in donkeys provides a number of benefits, including the ability to directly visualize the needle and distribute local anesthetic and avoid unintentional vascular damage in comparison with traditional blind techniques. Most US-guided sacrococcygeal space injections are straightforward techniques that are easy to learn and can be used in field conditions. Consequently, more research is required to assess this method in clinical cases.

**Conclusion**.

US-guided injection procedures revealed the performance time required for perfect placing the needle was significantly less than with a blind one. A shorter time for the onset of analgesia was achieved with the ultrasound-guided method, although the difference was not statistically significant. The ultrasound-guided epidural injection technique provided a number of benefits over the blind one, including the capacity to directly view the needle and distribute local anesthetic, avoid unintentional vascular damage, and quickly produce analgesia in comparison with traditional blind techniques. In conclusion, the ultrasound-guided epidural injection technique provides enhanced visualization of anatomical landmarks for accurate injection placement to offer efficient and safe anesthesia in the surgical approach in the sacrococcygeal region of the Egyptian donkeys.

## Data Availability

The datasets used and/or analyzed during the current study are available from the corresponding author on reasonable request.
